# Petaloid technique and prognostic significance of macular hole shapes by optical coherence tomography for full thickness macular hole

**DOI:** 10.3389/fmed.2024.1424580

**Published:** 2024-09-23

**Authors:** Khusbu Keyal, Bing Li, Chunyu Liu, Zhongping Tian, Houshuo Li, Yanlong Bi

**Affiliations:** Department of Ophthalmology, Tongji Hospital, School of Medicine, Tongji University, Shanghai, China

**Keywords:** full thickness macular hole, internal limiting membrane, optical coherence tomography, petaloid technique, pars plana vitrectomy

## Abstract

Surgical advancements for full-thickness macular hole (FTMH) treatment include vitrectomy, membrane peeling, and the inverted flap technique (IFT). IFT, which involves inverting the internal limiting membrane (ILM) flap over the macular hole (MH) or into the MH, improves success rates and visual recovery. However, issues like mis-aspiration during flap handling have been problematic. We introduce the petaloid technique, to position the ILM flap under air during FTMH surgery to evaluate its outcomes. This retrospective study included 28 eyes, with a mean minimum linear diameter (MLD) 472.04 ± 199.7 μm and basal diameter (BD) of 834.95 ± 593.54 μm. Primary closure of MH was achieved in 96.42% of patients, with 3.57% showing persistent MH during the 6-month follow-up. The VA improved significantly from preoperative levels at each postoperative stage, with notable increases at 3 months (0.86 ± 0.49 logMAR; *p* = 0.0132) and 6 months (0.77 ± 0.41 logMAR; *p* = 0.000081). The new closure patterns showed Type A in 28.6%, B in 14.3%, C in 28.6%, and D in 25%. Among different types of closure patterns, although VA improved in all types, significant improvement in VA was noted for type A and type C, with notable improvements at the 6-month follow-up for Type A (0.60 ± 0.23 logMAR; *p* = 0.02) and at the 1-month follow-up for Type C (0.62 ± 0.28 logMAR; p = 0.02). For macular hole retinal detachment in 5 eyes, the average preoperative VA was 1.86 ± 0.19 logMAR, while the final mean postoperative VA showed a significant improvement to 1.1 ± 0.40 logMAR (*p* = 0.021, paired *t*-tests). Retinal reattachment was achieved in all cases (5/5) without recurrent detachment post-silicone oil removal. For the prognostic significance of MH shapes identified by OCT, it can be concluded that the shape of macular holes significantly influences visual acuity outcomes at 6 months post-surgery (*p* = 0.037). The shape of macular holes, particularly Flask-shaped, significantly impacts visual acuity compared to other shape. The modified petaloid technique for treating FTMH proved safe and effective, with no significant complications noted.

## 1 Introduction

Before the introduction of vitrectomy to treat MH, the spontaneous closure rate for stage 2 MHs was 11.4% while for Gass stage 3 and 4 MHs it was 4%, (1). Kelly and Wendel (2) proposed vitrectomy as a treatment for MH, which resulted in 58% closure rate. As surgical techniques and instrumentation have improved, the success rates for the closure of macular holes have now exceeded 90% (3). So, surgery remains the primary treatment modality for FTMH, with ongoing advancements aimed at refining surgical outcomes and minimizing complications. Over time, in conjunction with pars plana vitrectomy (PPV), a spectrum of adjuvant and modified techniques has been integrated. Noteworthy innovations include ILM peeling, introduced by Guber et al. (4), extended ILM peeling by D’Souza et al. (5), autologous retinal grafting by Grewal et al. (6), the use of whole blood or blood components to improve macular hole bridging by Tam et al. (7), the use of amniotic membrane by Rizzo et al. (8), macular subretinal space injection of fluid for MH edge rim approximation by Meyer et al. (9), superior wide-based ILM flap transposition by Tabandeh et al. (10), neurosensory retina transplantation by Patel et al. (11), and the utilization of vitreous substitutes by Baino (12). With the continuous advancements in surgical techniques, these has enhanced the closure rate and the rate of visual acuity recovery across all types of MH. However, challenges were encountered after the flap was made; it was easy for the flap to be mis-aspirated by the flute needle or by the vitrectomy tip during fluid air exchange.

The aim of our study, using a 25-gauge PPV system, is to present a modified technique, the petaloid technique with repositioning of ILM flap into the MH under air for all types of FTMH. The main objectives included achieving anatomical closure of MH as verified by OCT after a single procedure. Secondary objectives encompassed assessing the time taken for complete closure of the MH and the extent of improvement in BCVA at the end of the follow-up period.

## 2 Methods

### 2.1 Preoperative preparations

This retrospective study was approved by the Research Ethics Committee of Tongji Hospital affiliated with Tongji University School of Medicine and conducted in accordance with the Declaration of Helsinki. All patients provided informed consent for the procedure and subsequent clinical monitoring. The study enrolled patients who underwent FTMH surgery at our hospital between January 2022 and November 2023. Inclusion criteria included confirmed FTMH diagnosis using an indirect ophthalmoscopy and OCT, along with symptomatic complaints of decreased visual acuity (VA) and metamorphopsia. Exclusion criteria included retinal diseases (age-related macular degeneration, diabetic retinopathy), other causes of decreased vision (corneal scarring, glaucoma with absolute visual field defects), prior surgical history, and incomplete medical records. Baseline demographic information, including gender, age, and FTMH duration, was collected. Preoperative assessments included best-corrected visual acuity (BCVA), slit-lamp biomicroscopy, intraocular pressure measurement (IOP), lens clarity evaluation, dilated indirect ophthalmoscopy, and OCT imaging (Zeiss Cirrus, HD-OCT, Model-5000). OCT scans were performed preoperatively and postoperatively. Macular hole closure was determined based on postoperative OCT findings. Follow-up evaluations were done at 1 week, 1 month, 3 months, and 6 months post-surgery.

### 2.2 Surgical technique

**Figure 1:**
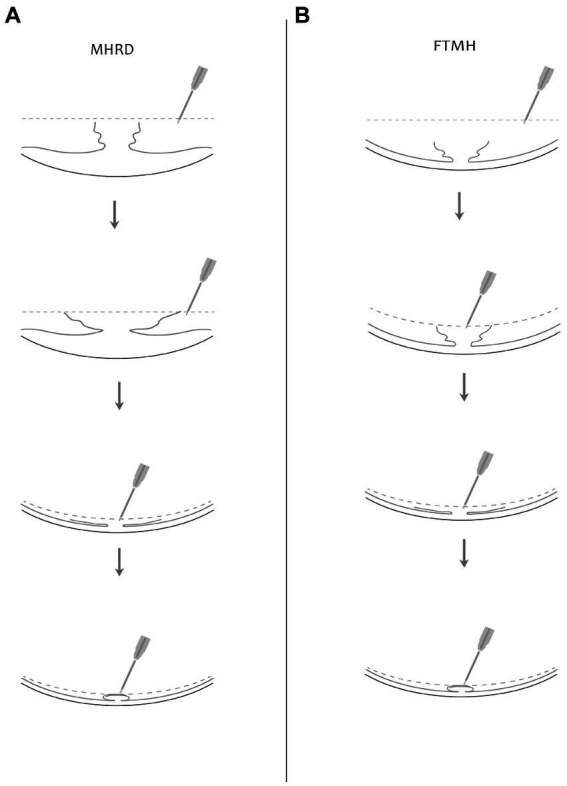
Illustrated here is a diagram showcasing the surgical procedure involving fovea-sparing ILM peeling, coupled with the repositioning of the ILM flap under air. Two scenarios are depicted: **(A)** for macular hole retinal detachment (MHRD) and **(B)** for full-thickness macular hole (FTMH). The dotted line in the figure indicates the air level. The instrument utilized is a flute needle, with different configurations shown in the figures to depict various stages of flap manipulation. In the second figure, the mouth of the flute needle is fully open, while in the third figure, it is partially open, and in the fourth figure, it is fully closed to prevent mis-aspiration of the ILM flap. Repositioning of the ILM flap is demonstrated under air, as depicted in the fourth figure.

A 25-gauge three port PPV was conducted utilizing the Alcon Constellation Vision System, with all procedures being performed by Dr. Bi Y. Patients were administered retrobulbar anesthesia composed of a mixture of 2% lidocaine and 0.75% bupivacaine. Standard pars plana core vitrectomy was performed aided by triamcinolone acetate for improved visualization of the vitreous. Staining of the ILM was achieved by applying Brilliant blue G dye for a duration of 20 s. The ILM peeling was conducted in two phases: initially focusing on the perifoveal area and extending radially to encompass the upper and lower vascular arcades, including both temporal and nasal regions. Subsequently, a second phase involved sparing the fovea during ILM peeling, creating a 360-degree ILM flap that remained attached to the rim of the macular hole, approximately 200 μm around the fovea. The elevated ILM flap was then trimmed into small flaps using a vitrectomy cutter, thus forming a petaloid shape ILM, followed by a complete fluid-air exchange. One needs to be cautious during this step, as excessive suction by the cutter could result in the loss of the flap. Repositioning of the petaloid shape ILM flap over the center of the macular hole according to MH size was done under air using a soft silicone-tipped draining flute needle. Careful adjustments were made to prevent fluid-induced floating of the ILM during this process by manipulating the orientation of the needle and gradually draining the fluid. To mitigate the risk of inadvertent ILM aspiration, air pressure was reduced to approximately 15 mmHg (Figure 1). Postoperatively, patients were advised to lie on their lateral side initially to prevent fluid accumulation in the macular area. For those with a clear lens, a strict prone position was recommended to prevent the formation of posterior subcapsular opacity. Sterilized air tamponade was used for 23 patients, whereas silicone oil (SiO) was used in five patients with macular hole retinal detachment (MHRD). In cases where significant lens opacification hindered fundus visualization during vitrectomy, small incision phacoemulsification cataract surgery with intraocular lens implantation was performed prior to vitrectomy. Combined cataract surgery was conducted in 22 patients.

MHs were categorized based on the International Vitreomacular Traction Study Group Classification into small (<250 μm), medium (250–400 μm), or large (>400 μm), with or without persistent VMT (13). Measurements were conducted utilizing the OCT caliper function for both the minimum linear diameter (MLD) at the narrowest part of the hole and the base diameter (BD) parallel to the RPE at the nearest point of retinal apposition. VA was documented in decimal values and then converted into logarithm of minimal angle of resolution (logMAR) units for statistical analysis, with non-numeric values adjusted accordingly: count fingers = 1.7 logMAR, hand movement = 2.0 logMAR (14). Statistical analyses were carried out using SPSS version 28.0 (SPSS Inc., Chicago, IL, United States), representing continuous variables as mean ± standard deviation (SD) and categorical variables as percentages. An independent-sample *t*-test was utilized to compare parameters pre-surgery and at subsequent follow-up intervals, with a *p* < 0.05 indicating statistical significance. Furthermore, the postoperative closure type of the macular hole and its correlation with the degree of postoperative visual improvement were statistically examined.

## 3 Results

### 3.1 Clinical characteristics of the patients

Patient demographics and clinical characteristics are summarized in Table 1. A cohort of 28 patients, with an average age of 67.03 ± 11.8 years, was included in this study, all diagnosed with FTMH. Among these patients, 20 presented with idiopathic or primary FTMH, while eight had secondary FTMH. Within the secondary FTMH group, five patients exhibited MHRD, 1 had a traumatic macular hole (TMH), and two had high myopia macular hole (HMMH). The gender distribution consisted of 10 males and 18 females. The mean duration of symptoms was 168.5 ± 267.5 days. The right eye was affected in 14 patients, while the left eye was affected in the other 14. On average, the minimum linear diameter measured 472.04 ± 199.7 μm, and the basal diameter was 834.95 ± 593.54 μm. According to the IVTS classification, 17 FTMH were classified as large (16 without vitreomacular traction (VMT) and 1 with VMT), while six were categorized as small (four without VMT and two with VMT). Additionally, five patients were diagnosed with MHRD associated with myopic MH, indicative of highly myopic eyes (beyond-6.0 diopters) with an average axial length of 28.5 ± 1.8 mm (range 27.0–31.5 mm).

**Table 1:** Clinical characteristics of 28 patients with FTMH.

Variables	Mean ± SD (Range)/*n*
Age (years)	67.03 ± 11.8 (16–82)
Gender (%)	
Male	10 (35.7%)
Female	18 (64.3%)
Laterality (%)	
Right	14 (50%)
Left	14 (50%)
Disease duration (days)	168.5 ± 267.5 (2–1,095)
Diagnosis	
IMH	20
HMMH	2
TMH	1
MHRD	5
Surgical procedure	
PPV+ Phaco+ IOL	22
PPV	6
Tamponade	
Air	23
Silicon oil	5
Macula hole size (μm)	472.04 ± 199.7 (143–806)
Base diameter	834.95 ± 593.54 (180–3,000)
IVTS classification	
Large w/o VMT	16
Small w/o VMT	4
Large w VMT	1
Small w VMT 2 New closure pattern	
Type A	28.6%
Type B	14.3%
Type C	28.6%
Type D	25%

SD, standard deviation; IMH, idiopathic macular hole; HMMH, high myopia macular hole; TMH, traumatic macular hole; MHRD, macular hole retinal detachment; PPV, pars plana vitrectomy; Phaco + IOL, phacoemulsification with intraocular lens; IVTS, classification-International vitreomacular traction study; w/o VMT, without vitreomacular traction; w, with vitreomacular traction.

### 3.2 New macular hole closure pattern based on OCT and MH closure rate

**Figure 2:**
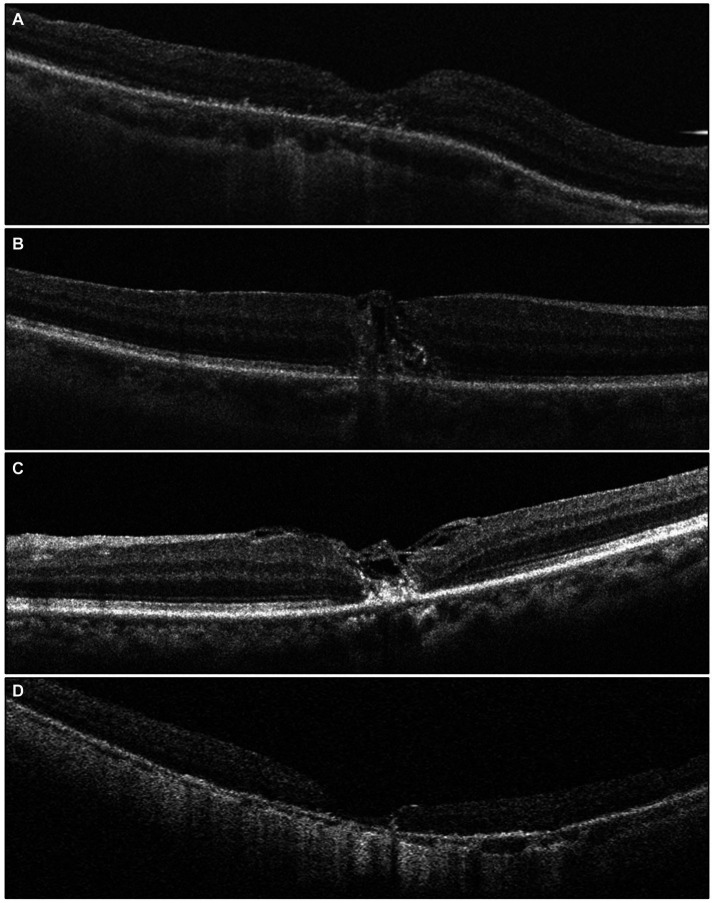
The grading of post-operative macular hole closure based on OCT images, reflecting the anatomical outcome, is as follows: **(A)** a foveal contour that appears normal, covering the retinal pigment epithelium (RPE) completely with closure of the macular hole (MH). **(B)** Irregular closure of the MH with tissue filling inside MH and an interrupted foveal contour. **(C)** Presence of bridging tissue still connected to the edges of the MH, resulting in MH closure. **(D)** The edges of the MH are attached to the RPE, indicating MH closure, but the fovea appears thinner with a loss of the ellipsoid zone.

Different modified surgical techniques lead to various morphologies of macular hole closure and wound healing, suggesting a need for reclassification of closure patterns. The closure of the MHs is characterized by the absence of retinal pigment epithelium (RPE) exposure to the vitreous chamber with uninterrupted tissue. The closure pattern of the MHs is described based on the contour of the fovea and the status of the MH edge rim. Type A-normal appearing foveal contour completely covering the RPE with closure of the macular hole. Type B-irregular closure of the macular hole with tissue filling in the macular hole and interrupted foveal contour. Type C-bridging tissue which is still attached to the edges of macular hole with closure of the macular hole. Type D-edges of the macular hole are attached to RPE with closure of the MH but the fovea is thinner with loss of the ellipsoid zone. Type A was found in 28.6%, Type B was found in 14.3%, Type C was found in 28.6% and Type D was found in 25%. Figure 2 illustrates the post-operative MH closure pattern observed in OCT. Initially, the MH closure rate was 96.42% (27 out of 28 eyes), with only 3.57% (1 out of 28 eyes) exhibiting persistent MH after an average follow-up period of 6 months. Notably, there were no instances of MH reopening throughout the 6-month follow-up period.

### 3.3 Functional outcome

**Figure 3:**
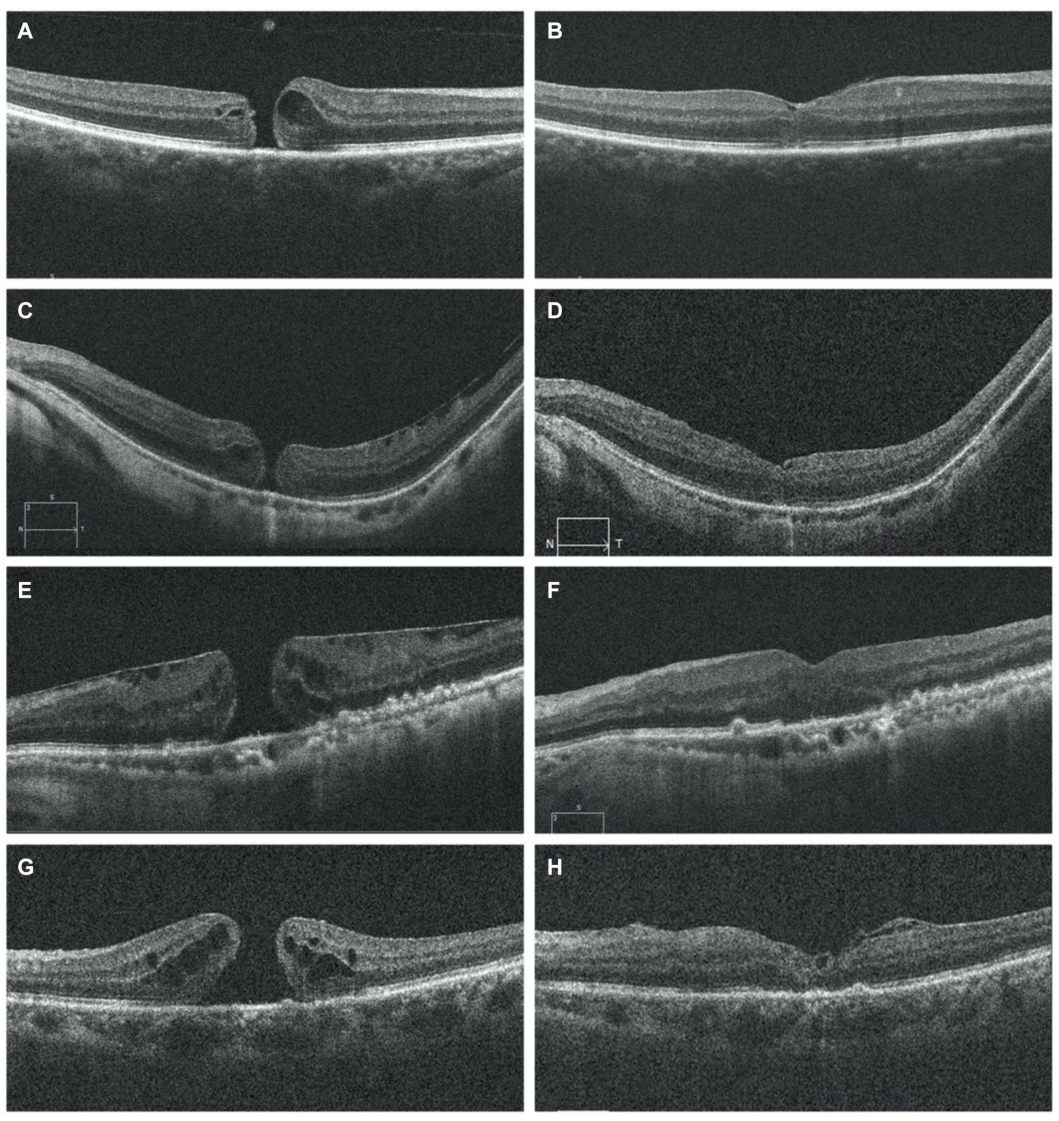
OCT image for FTMH preoperative on the left side **(A,C,E,G)** and postoperative on the right side **(B,D,F,H)**. OCT scans show the resolution of macular hole in follow-up period **(B,D,F,H)** MH was closed and the inverted ILM flap could be observed covering the hole with a hyperreflective tissue **(B,D,H)**. I At 6months postoperatively, recomposition of outer retinal layers (ELM and EZ were partially restored) but not completely restored. Six months after surgery, the hyperreflective foveal tissue were thickened on the surface of the retina in the parafovea area and was stable and the defects of ELM and EZ lines were persistent **(H)**. ILM, internal limiting membrane; ELM, external limiting membrane; EZ, ellipsoid zone; OCT, optical coherence tomography MH: macular hole. The presence of ILM remnants on the retinal surface was defined as hyperrefractive tissue.

Functional outcomes were assessed at 1, 3, and 6 months post-surgery to evaluate treatment responses. Preoperative and postoperative OCT pictures are shown in Figure 3. The VA increased gradually from the preoperative stage at each postoperative follow-up, with significant increases noted at the third month (0.86 ± 0.49 logMAR; *p* = 0.0132) and 6 months (0.77 ± 0.41 logMAR; *p* = 0.000081) (Table 2). Among different types of closure patterns, although VA improved in all types, significant improvement in VA was noted for type A and type C, with notable improvements at the 6-month follow-up for Type A (0.60 ± 0.23 logMAR; *p* = 0.02) and at the 1-month follow-up for Type C (0.62 ± 0.28 logMAR; p = 0.02) (Table 3). For MHRD in 5 eyes, the average preoperative VA was 1.86 ± 0.19 logMAR, while the final mean postoperative VA showed a significant improvement to 1.1 ± 0.40 logMAR (*p* = 0.021, paired *t*-tests).

**Table 2:** VA and IOP pre and post operation for FTMH.

Variables	Pre-op	Post-op	Post-op1m	Post-op3m	Post-op6m
IOP	14.6 ± 2.7	15.8 ± 3.2	–	–	–
LogMAR	1.10 ± 0.43	1.10 ± 0.55	0.98 ± 0.51	0.86 ± 0.49*	0.77 ± 0.41**

**p < 0.01, *p < 0.05, IOP, intraocular pressure; LogMAR, logarithm of the minimum angle of resolution.

**Table 3:** Types of macular hole closure pattern after operation for FTMH.

New closure pattern	Pre-op LogMAR	Post-op LogMAR	Post-op1M LogMAR	Post-op3M LogMAR	Post-op6M LogMAR
Type A	1.025 ± 0.381	0.825 ± 0.341	0.812 ± 0.422	0.637 ± 0.307	0.600 ± 0.239*
Type B	1.35 ± 0.332	1.375 ± 0.287	1.225 ± 0.287	0.975 ± 0.236	0.975 ± 0.236
Type C	0.962 ± 0.192	0.712 ± 0.391	0.625 ± 0.287*	0.587 ± 0.259*	0.500 ± 0.233*
Type D	1.2 ± 0.7	1.571 ± 0.525	1.442 ± 0.602	1.371 ± 0.642	1.157 ± 0.509

*p < 0.05, M, month; LogMAR, logarithm of the minimum angle of resolution.

### 3.4 Analyze the prognostic significance of macular hole shapes identified on OCT

**Figure 4:**
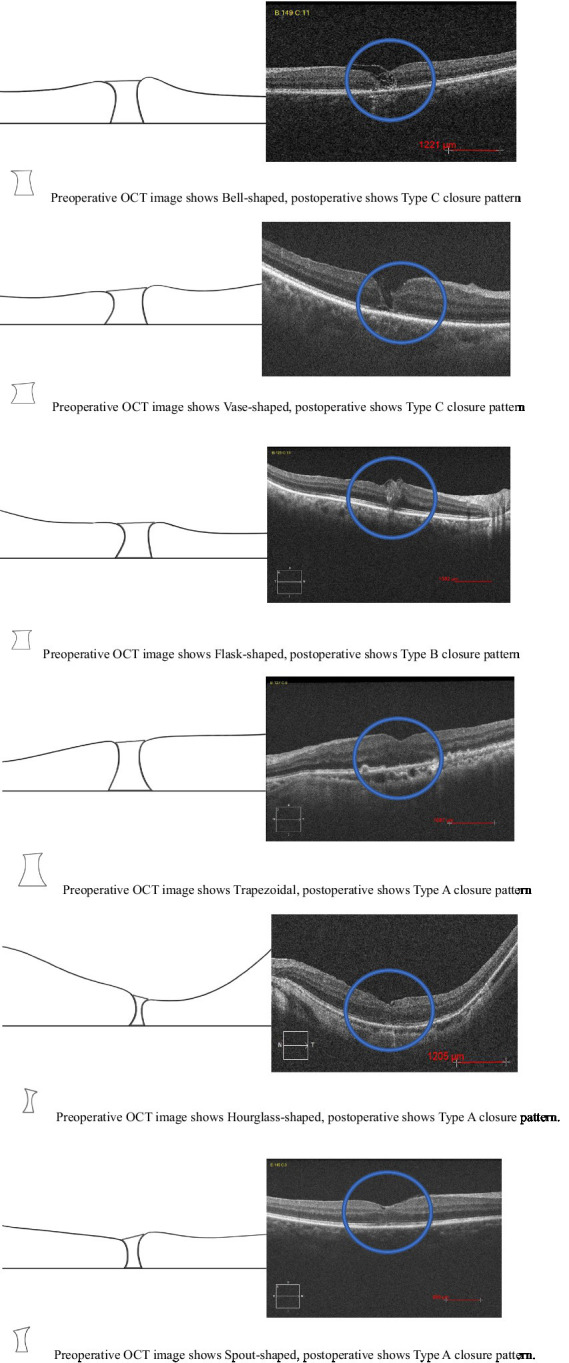
Preoperative OCT image shape of macular hole and postoperative new closure pattern for FTMH.

In our study, describing the shape of a macular hole as a prognostic factor, we considered using the shape of a macular hole as viewed on OCT preoperative as a new prognostic factor. These shapes are likely schematic representations to aid in the visualization and discussion of macular hole characteristics. This type of illustration can be used to help explain the anatomical features of a macular hole and identify a new indicator for the prognosis of the disease. The prognosis for vision depends significantly on the hole’s characteristics, size, duration and VMT: larger and longer-standing holes generally have a poorer prognosis, and the presence of VMT and its degree can affect both the shape of the hole and the surgical outcome. We developed a hypothesis regarding how different shapes could influence the prognosis and correlated the shapes with surgical outcomes such as anatomical closure and postoperative visual outcomes and the need for additional surgeries. The macular holes based on the OCT classification are described as having the following shapes: flask, bell, vase, trapezoidal, hourglass, and spout (Figure 4). There is a statistically significant difference in mean logMAR visual acuity among the different macular hole shapes at the 6-month postoperative period (ANOVA test, *p* = 0.031). This implies that the shape of a macular hole may have prognostic significance in terms of visual outcomes after treatment. The Tukey HSD post-hoc analysis provides comparisons between each pair of macular hole shapes. There is a statistically significant difference in the mean 6-month postoperative logMAR visual acuity between the “Flask” and “Vase” shapes (*p* = 0.0378). Specifically, “Flask”-shaped macular holes may be associated with better visual recovery compared to “Vase”-shaped holes. All other comparisons with “Bell,” “Trapezoid,” “Spout,” and “Hourglass” shapes did not show a statistically significant difference in visual acuity (*p* > 0.05). The macular hole shapes among the 28 eyes in our study is as follows: six eyes (21.42%) were flask-shaped, four eyes (14.28%) were vase-shaped, four eyes (14.28%) were bell-shaped, two eyes (7.14%) were trapezoid-shaped, three eyes (10.71%) were hourglass-shaped, and three eyes (10.71%) were spout-shaped. These results suggest that the shape of the macular hole might influence the visual outcome, with “Flask” and “Vase” shapes having the most significant difference in postoperative visual acuity and it can be particularly useful when considering the prognosis of macular hole surgeries. However, due to the limitations observed in the statistical tests, primarily because of possible insufficient sample sizes or lack of variance in some categories, these findings should be interpreted with caution. Further study with a larger dataset that ensures a sufficient number of cases in each category could provide more definitive conclusions. Additionally, a multidimensional approach that considers other factors, such as the size of the hole, the duration before surgery, and patient-specific characteristics, would offer a more comprehensive understanding of prognostic factors. Further studies are recommended to validate these results and potentially refine patient management protocols accordingly.

### 3.5 Follow up

**Figure 5:**
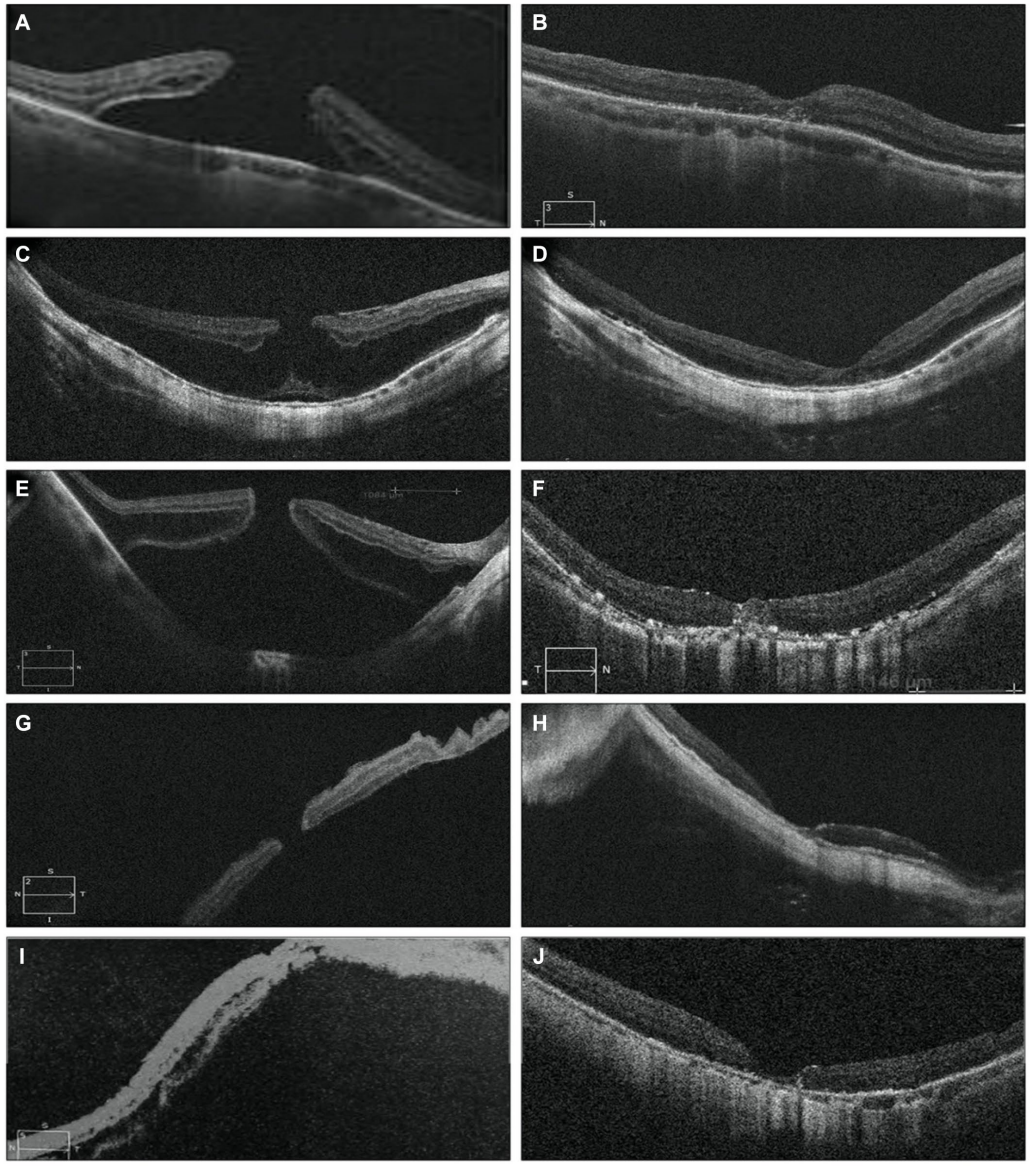
Pre-operative OCT images of five eyes with MHRD are depicted on the left side in **(A,C,E,G,I)**, which confirms the macular hole retinal detachment. While post-operative OCT images are shown in **(B,D,F,H)** and **(J)** shows MH closure and retinal reattachment without residual SRF and with thickening of the fovea and partial recovery of the ellipsoid zone at the perifoveal area. **(B)** Shows the OCT scan of a 76-year-old male’s right eye taken 3 weeks postoperatively, while **(D)** displays the OCT scan of a 65-year-old male’s right eye taken 1 month postoperatively. In **(F)**, the OCT scan of a 68-year-old female’s right eye taken 2 weeks postoperatively reveals the remnant of the internal limiting membrane (ILM) flap filling the MH, providing a scaffold for retinal layer growth and indicating partial restoration of the normal macular profile. **(H)** Depicts the OCT scan of a 69-year-old male’s left eye taken 1 week postoperatively, and **(J)** shows the OCT scan of a 68-year-old female left eye taken 2 months postoperatively. In **(H,J)** there is post operatively decrease in central foveal thickness.

Postoperative follow-up was scheduled at intervals of 1 week, 1 month, 3 months, and 6 months. These assessments included measurement of BCVA, IOP monitoring, fundus examinations, and OCT scans. The removal of SiO was scheduled for at least 3 months after the initial surgery for five patients. In MHRD the initial rate of successful anatomical reattachment was 100% (5 out of 5 cases), and this attachment was maintained throughout the duration of the final follow-up visit, which occurred at 6 months without any instances of reopening. OCT revealed a normal concave macular configuration in all highly myopic eyes postoperatively, with firm attachment of MH margins to the underlying RPE (Figure 5). Recurrent retinal detachment was not observed after silicone oil removal in all cases.

## 4 Discussion

The inverted flap technique, involving the peeling and inversion of the ILM into or over the MH, has evolved over time. Various modifications have been introduced, such as the use of viscoelastic and perfluorocarbon liquid to prevent flap displacement, as well as different flap configurations including temporal-only, single-layered, semi-circular, and cabbage-leaf techniques. Additionally, alternative tissues such as autologous retinal flap, human amniotic membrane, and the anterior or posterior lens capsule have been explored for hole filling (15). For instance, Michalewska et al. (16) demonstrated a temporal flap technique with a 93% closure rate, although flap retroversion during air–fluid exchange resulted in surgical failure in two cases necessitating reoperation. Since then, the inverted ILM flap technique has gained popularity for large MHs, significantly improving closure rates postoperatively, although it has drawbacks such as potential flap detachment during fluid-air exchange (17). The employment of an inverted ILM flap, typically utilized for chronic or large MH, has demonstrated a higher closure rate in myopic MH cases (18, 19), albeit encountering challenges with flap unfolding. Inserting an ILM flap into the hole was found to mitigate the risk of the flap flipping back (20); however, this method poses a potential risk of the inserted tissue interfering with foveal neuroretinal tissue and causing damage to the RPE (21). Shin et al. (22) employed perfluorooctane in conjunction with single-layer ILM stabilization during air–fluid exchange; however, this technique necessitated additional manipulation, and in one instance, the flap still displaced, resulting in failure to close the hole. Chen et al. (23) used a modified technique where, following partial air–fluid exchange, the gravity effect of retained vitreous fluid facilitated the descent of the ILM flap to cover the macular hole once the patient assumed a sitting position. In our study, we implemented a modified approach with a broad 360° ILM flap adhering to the base, aiming to resist ILM misaspiration. Repositioning the ILM under air ensured adequate coverage of the MH center. OCT examinations post-surgery and during follow-up visits confirmed the ILM’s positioning over the fovea, suggesting a potentially effective strategy to mitigate complications associated with the inverted flap technique. In our study, patients with high myopia macular holes were included and exhibited enhanced postoperative visual and anatomical outcomes.

MHRD often occurs in highly myopic eyes, particularly those with posterior staphyloma. Existing research suggests that even with successful retinal reattachment, the macula typically does not fully regain its normal anatomical structure in highly myopic eyes. This lack of restoration may be due to the inherent shortness of the retina, which prevents complete coverage of the entire posterior segment despite successful retinal reattachment. Additionally, manipulation of the retina in highly myopic eyes poses a significant risk of complications due to its thin and fragile nature. Various factors, including chorioretinal atrophy at the posterior pole, the condition of the RPE, the morphology and size of the staphyloma, excessive axial elongation of the globe, and the mismatch between the elongated sclera and the extent of the retina, are identified as potential impediments to achieving anatomical success and closure of macular holes in highly myopic eyes. Recent advancements in the surgical management of MHRD in highly myopic eyes include the technique of employing an inverted ILM flap (24–26). In our study, in MHRD the initial rate of successful anatomical reattachment was 100% (5 out of 5 cases), and this attachment was maintained throughout the duration of the final follow-up visit, which occurred at 6 months without any instances of reopening.

The new postoperative OCT classification of MH closure patterns is a valuable tool for understanding the outcomes of surgical intervention and the healing process. In Types A and C, they maintain a foveal contour, with restoration of the inner retinal layers with significant improvement in VA. Tailoring surgical techniques and postoperative management to these different closure patterns could potentially enhance visual outcomes and patient satisfaction. We have also analyzed the prognostic significance of MH shapes identified by OCT in the preoperative period with distinctive shapes such as flask, bell, vase, trapezoidal, hourglass, and spout. Based on the results, it can be concluded that the shape of macular holes significantly influences visual acuity outcomes 6 months post-surgery. Notably, “Flask”-shaped macular holes are associated with better visual recovery compared to “Vase”-shaped holes. These findings suggest that the shape of macular holes, as assessed by OCT, could be an important factor in predicting the success of surgical interventions, may influence treatment strategies and play a significant role in predicting visual recovery. The modifications outlined in this study offer several advantages, including the use of air reduced prone positioning time for patients. Key procedural aspects included maintaining a clear posterior pole view. A broad 360° ILM flap adhered to the base, and the attached distance of approximately 200 μm around the fovea rim helped to resist ILM mis-aspiration. Repositioning the ILM under air helped ensure MH coverage and flap retention, preventing fluid-induced ILM floating. During repositioning, air pressure was reduced to minimize the risk of ILM mis-suction. Migration of ILM positioning into the hole was to allow tissue bridging and healing to occur with restoration of macular hole anatomy and no residual retinal defect. Patients’ heads were promptly turned to the lateral side postoperatively to prevent fluid-induced ILM floating.

## 5 Conclusion

This research introduces a modified technique, the petaloid technique with repositioning of the ILM flap into the MH under air for all types of FTMH. Furthermore, it proposes a postoperative OCT classification system for assessing MH closure patterns. Our study has also analyzed the prognostic significance of MH shapes identified by OCT in the preoperative period with distinctive shapes. Successful treatment of all types of FTMH was observed. Post-surgery OCT evaluations and follow-up visits confirmed ILM positioning over the fovea, with closure achieved for all FTMH types except one large MH without VMT. No other intraoperative or postoperative complications were observed. This surgery demonstrates a significant positive impact on visual acuity. These findings advocate for the continued use and development of surgical techniques to improve patient outcomes.

### 5.1 Limitations

Our study has several limitations, including its single-arm, retrospective nature, small sample size, and relatively short follow-up period. To gain a comprehensive understanding of the durability of the surgical benefits observed, future investigations could explore extending beyond the 6-month period. A larger dataset that ensures a sufficient number of cases in each category could provide more definitive conclusions. Additionally, a multidimensional approach to understanding prognostic factors is warranted to explore the underlying mechanisms influencing these differences in visual recovery.

## Data Availability

The raw data supporting the conclusions of this article will be made available by the authors, without undue reservation.

## References

[1] Wu TT Kung YH. Comparison of anatomical and visual outcomes of macular hole surgery in patients with high myopia vs. non-high myopia: a case-control study using optical coherence tomography. Graefes Arch Clin Exp Ophthalmol. (2012) 250:327–31. doi: 10.1007/s00417-011-1821-7, PMID: 21935606

[2] Kellyand NE Wendel RT. Vitreous surgery for idiopathic macular holes. Results of a pilot study. Arch Ophthalmol. (1991) 109:654–9. doi: 10.1001/archopht.1991.01080050068031 2025167

[3] la Cour M Friis J. Macular holes: classification, epidemiology, natural history and treatment. Acta Ophthalmol Scand. (2002) 80:579–87. doi: 10.1034/j.1600-0420.2002.800605.x, PMID: 12485276

[4] Guber J Lang C Valmaggia C. Internal limiting membrane flap techniques for the repair of large macular holes: a short-term follow-up of anatomical and functional outcomes. Klin Monatsbl Augenheilkd. (2017) 234:493–6. doi: 10.1055/s-0042-119694 28147400

[5] D’Souza MJ Chaudhary V Devenyi R Kertes PJ Lam WC. Re-operation of idiopathic full-thickness macular holes after initial surgery with internal limiting membrane peel. Br J Ophthalmol. (2011) 95:1564–7. doi: 10.1136/bjo.2010.195826, PMID: 21355018 PMC3199446

[6] Grewal DS Mahmoud TH. Autologous neurosensory retinal free flap for closure of refractory myopic macular holes. JAMA Ophthalmol. (2016) 134:229–30. doi: 10.1001/jamaophthalmol.2015.5237, PMID: 26720054

[7] Tam ALC Yan P Gan NY Lam WC. The current surgical management of large, recurrent, or persistent macular holes. Retina. (2018) 38:1263–75. doi: 10.1097/IAE.0000000000002020 29300247

[8] Rizzo S Caporossi T Tartaro R Finocchio L Franco F Barca F. A human amniotic membrane plug to promote retinal breaks repair and recurrent macular hole closure. Retina. (2019) 39:S95–S103. doi: 10.1097/IAE.0000000000002320 30312261

[9] Meyer CH Szurman P Haritoglou C Maier M Wolf A Lytvynchuk L. Application of subretinal fluid to close refractory full thickness macular holes: treatment strategies and primary outcome: APOSTEL study. Graefes Arch Clin Exp Ophthalmol. (2020) 258:2151–61. doi: 10.1007/s00417-020-04735-3, PMID: 32583283

[10] Tabandeh H Morozov A Rezaei KA Boyer DS. Superior wide-base internal limiting membrane flap transposition for macular holes: flap status and outcomes. Ophthalmol Retina. (2021) 5:317–23. doi: 10.1016/j.oret.2020.12.003, PMID: 33316462

[11] Patel SN Mahmoud TH Kazahaya M Todorich B. Autologous neurosensory retinal transplantation: bridging the gap. Retina. (2021) 41:2417–23. doi: 10.1097/IAE.0000000000003210, PMID: 33990116

[12] Baino F. Towards an ideal biomaterial for vitreous replacement: historical overview and future trends. Acta Biomater. (2011) 7:921–35. doi: 10.1016/j.actbio.2010.10.030, PMID: 21050899

[13] Duker JS Kaiser PK Binder S de Smet MD Gaudric A Reichel E. The international Vitreomacular traction study group classification of vitreomacular adhesion, traction, and macular hole. Ophthalmology. (2013) 120:2611–9. doi: 10.1016/j.ophtha.2013.07.042, PMID: 24053995

[14] Chng SW Patton N Ahmed M Ivanova T Baumann C Charles S. The Manchester large macular hole study: is it time to reclassify large macular holes? Am J Ophthalmol. (2018) 195:36–42. doi: 10.1016/j.ajo.2018.07.027 30071212

[15] Zhao PP Wang S Liu N Shu ZM Zhao JS. A review of surgical outcomes and advances for macular holes. J Ophthalmol. (2018) 2018:1–10. doi: 10.1155/2018/7389412 PMC5932482 29850211

[16] Michalewska Z Michalewski J Dulczewska-Cichecka K Adelman RA Nawrocki J. Temporal inverted internal limiting membrane flap technique versus classic inverted internal limiting membrane flap technique: a comparative study. Retina. (2015) 35:1844–50. doi: 10.1097/IAE.0000000000000555, PMID: 25946691

[17] Michalewska Z Michalewski J Adelman RA Nawrocki J. Inverted internal limiting membrane flap technique for large macular holes. Ophthalmology. (2010) 117:2018–25. doi: 10.1016/j.ophtha.2010.02.011 20541263

[18] Kuriyama S Hayashi H Jingami Y Kuramoto N Akita J Matsumoto M. Efficacy of inverted internal limiting membrane flap technique for the treatment of macular hole in high myopia. Am J Ophthalmol. (2013) 156:125–31. doi: 10.1016/j.ajo.2013.02.014, PMID: 23622567

[19] Michalewska Z Michalewski J Dulczewska-Cichecka K Nawrocki J. Inverted internal limiting membrane flap technique in macular hole associated with pathological myopia. Retina. (2014) 34:664–9. doi: 10.1097/IAE.0000000000000042, PMID: 24263468

[20] Chen SN Yang CM. Inverted internal limiting membrane insertion for macular hole-associated retinal detachment in high myopia. Am J Ophthalmol. (2016) 162:99–106. doi: 10.1016/j.ajo.2015.11.013, PMID: 26582311

[21] Theodossiadis GP Chatziralli IP Theodossiadis PG. Inverted internal limiting membrane insertion for macular hole-associated retinal detachment in high myopia. Am J Ophthalmol. (2016) 165:206–7. doi: 10.1016/j.ajo.2016.03.002, PMID: 27041100

[22] Shin MK Park KH Park SW Byon IS Lee JE. Perfluoro-n-octane-assisted single-layered inverted internal limiting membrane flap technique for macular hole surgery. Retina. (2014) 34:1905–10. doi: 10.1097/IAE.0000000000000339, PMID: 25154029

[23] Chen SN. Large semicircular inverted internal limiting membrane flap in the treatment of macular hole in high myopia. Graefes Arch Clin Exp Ophthalmol. (2017) 255:2337–45. doi: 10.1007/s00417-017-3808-5, PMID: 28993905

[24] Chatziralli I Machairoudia G Kazantzis D Theodossiadis G Theodossiadis P. Inverted internal limiting membrane flap technique for myopic macular hole: a meta-analysis. Surv Ophthalmol. (2021) 66:771–80. doi: 10.1016/j.survophthal.2021.02.010, PMID: 33652002

[25] Matsumura T Takamura Y Tomomatsu T Arimura S Gozawa M Kobori A. Comparison of the inverted internal limiting membrane flap technique and the internal limiting membrane peeling for macular hole with retinal detachment. PLoS One. (2016) 11:e0165068. doi: 10.1371/journal.pone.0165068, PMID: 27764184 PMC5072623

[26] Takahashi H Inoue M Koto T Itoh Y Hirota K Hirakata A. Inverted internal limiting membrane flap technique for treatment of macular hole retinal detachment in highly myopic eyes. Retina. (2018) 38:2317–26. doi: 10.1097/IAE.0000000000001898, PMID: 29065014

